# What cooling pond sediments can reveal about ^14^C in nuclear power plant liquid effluents: Case study Lake Drūkšiai, Ignalina nuclear power plant cooling pond

**DOI:** 10.1371/journal.pone.0285531

**Published:** 2023-10-20

**Authors:** Rūta Barisevičiūtė, Jonas Mažeika, Evaldas Maceika, Laurynas Juodis, Vytautas Rakauskas, Olga Jefanova, Žilvinas Ežerinskis, Justina Šapolaitė, Laurynas Butkus, Vidmantas Remeikis

**Affiliations:** 1 State Research Institute Center for Physical Sciences and Technology, Vilnius, Lithuania; 2 State Research Institute Nature Research Centre, Vilnius, Lithuania; University of Florida, UNITED STATES

## Abstract

The vertical distribution of radiocarbon (^14^C) was examined in two bottom sediment cores, taken from Lake Drūkšiai which had been used as a cooling pond for the Ignalina nuclear power plant (INPP) with two RBMK type reactors. The aim of this work was to reconstruct ^14^C amounts in the lake ecosystem during an 8-year period after the INPP was closed, as any official monitoring of ^14^C in liquid releases from the INPP was not performed. The possibility of comparing the variation of the ^14^C specific activity in the corresponding layers of the same period of 3 different cores (one taken in 2013 and two in 2019) revealed the variability of the determined values of liquid radiocarbon discharges from the INPP into the lake. Cores taken in 2019 showed a permament^14^C release rate of 0.76±0.06 GBq/y all eight years after the closure of the INPP. The ^14^C release rate established from radiocarbon measurements in both cores did not differ by more than 0.8 GBq/y. However, including data from the core taken several years ago, the estimated radiocarbon release rate values varied within 1.3 GBq/y.

## Introduction

Radiocarbon is produced in nuclear reactors through neutron-induced reactions with ^14^N, ^17^O, and ^13^C isotopes present in the materials of fuel, moderator and coolant of reactors [1]. Areas in close proximity to nuclear power plants are most affected by airborne releases. Normally, liquid effluents are considered to contain relatively low amounts of ^14^C. It was determined that ^14^C in liquid effluents from Light Water Reactors (pressure water (PWRs) and boiling water (BWRs) reactors) constitute less than 1% (with the exception of Indian Point PWR, where it was estimated to be approximately 1.8% [2]) of atmospheric releases [1–3].

The Ignalina nuclear power plant (INPP) operated two RBMK-1500 units (design electric power 1500 MW_e_ and decreased to 1350 MWe due to safety concerns after the Chernobyl accident). The RBMK (Russian acronym for ‘Channelized Large Power Reactor‘) is a graphite-moderated boiling water channel-type reactor with the same principle of electricity generation as the BWRs. An official monitoring of ^14^C in liquid releases from the Ignalina nuclear power plant was never performed. Any official data concerning liquid ^14^C releases from the other RBMK-type reactors are not available. However, the INPP was one of the rarer cases when the nuclear power plants that used the lake as a pond for cooling and industrial discharges. Furthermore, the relatively long water residence time in the lake (of 3 y) allowed reconstruction of the routine ^14^C liquid releases from the INPP to its cooling pond Lake Drūkšiai with sufficiently high temporal resolution by analysing the radiocarbon vertical distribution in sediment core samples, taken in the main sedimentation area of the lake [4].

In our previous work [5, 6], we evaluated ^14^C liquid discharges from INPP over its entire operation period by analyzing radiocarbon concentration variation in two organic sediment fractions: alkali- soluble and -insoluble. The sediment core was taken in 2013, and covered only a four-years period after the closure of the INPP. During this period, the estimated ^14^C excess in lake sediment organic matter [6] was a few times higher than that recorded during the first 14 years of the INPP operation. Furthermore, our previous studies on ^14^C airborne releases from INPP, when radiocarbon measurements were performed in tree rings of pine trees sampled at a distance of 6.6 km from the nuclear power plant, showed up to 5–6% higher ^14^C concentrations (compared to samples from the background site) during 4–5 years after plant closure. Similar ^14^C increases in tree rings were observed during the first 14 years of INPP operation (1984–1997), when only minor maintenance activities in the INPP were performed [7–9]. Therefore, there was a need to check ^14^C liquid effluents from the INPP to the lake for the longer period after the final shutdown of both reactors. Furthermore, no information is available in open sources on the liquid ^14^C releases of the NPP with RBMK reactors in the decommissioning stage under the ongoing dismantling activities of the reactor’s constructions, systems, etc.

Therefore, the aim of this work was to trace ^14^C amounts in the lake ecosystem after the INPP was closed in 2009.

To cope with the aim of the study, two sediment cores sampled in 2019 were analyzed. Additionally, the pelagic and benthivorous fish scales caught in 1980–1999 and 2005–2019 were measured (the data of the *Coregonus albula* scales from the periods 1980–1999 and 2005–2012 were taken from [5]).

In this work, we had the possibility to compare the radiocarbon distribution in three sediment cores (the core sampled in 2013 (data taken from [5]) and 2 cores in 2019). The data of radiocarbon specific activity variations between layers of several cores corresponding to the same period showed significant variability in determining ^14^C in liquid releases using this method.

## Materials and methods

### Site description

**Fig 1:**
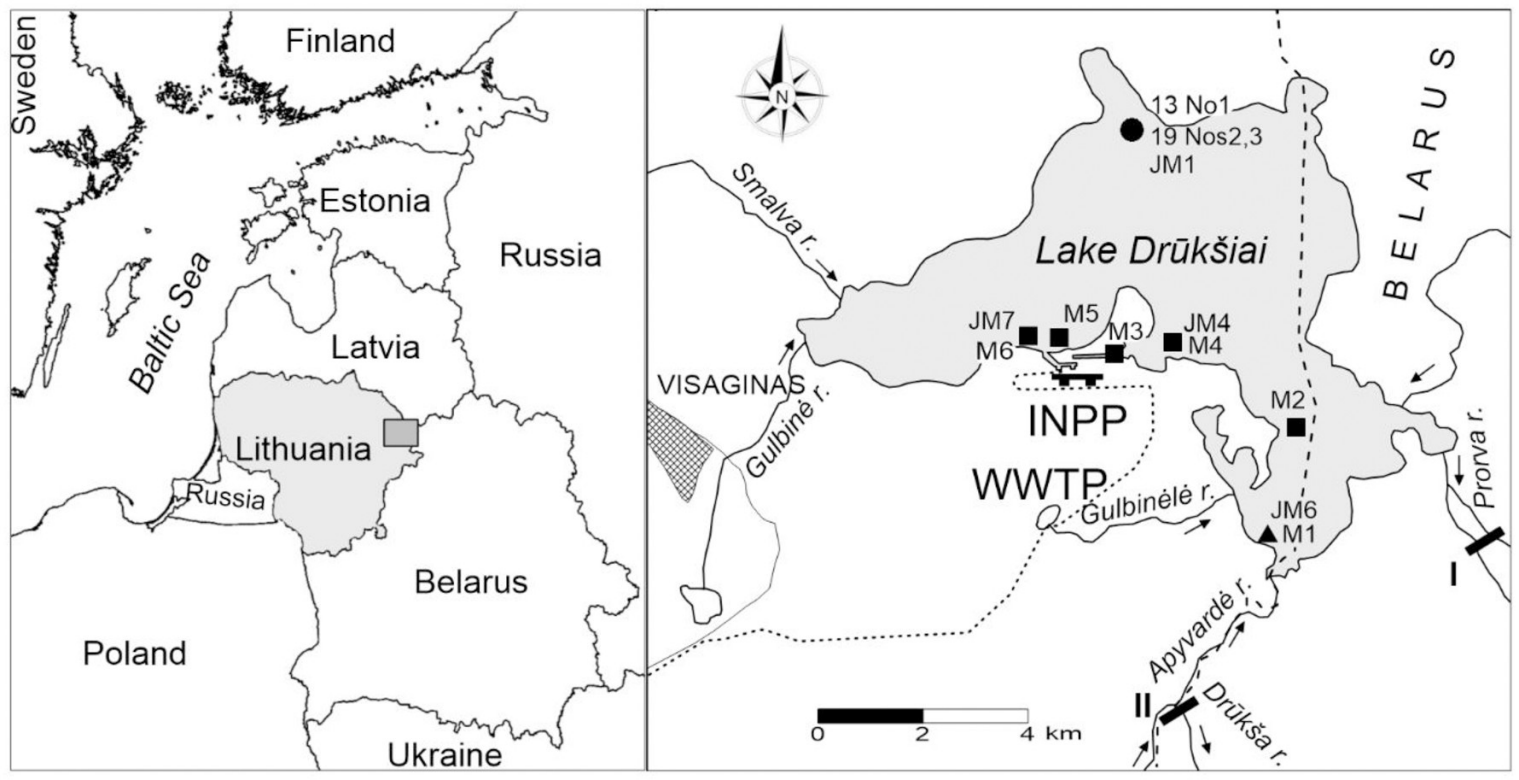
Location of the study area with sampling sites in Lake Drūkšiai. The sediment cores, DIC, and aquatic plant sampling sites are marked by a circle, triangle, and squares, respectively. The hydroelectric power plant (HEPP) and the dam on Drūkša river are marked by black bars I and II, respectively. WWTP means the location of waste water treatment plant nearby a small lake connected to Lake Drūkšiai by the Gulbinėlė streamlet. The industrial rain drainage system (IRD) of Visaginas town via the Smalva and Gulbinėlė streams is connected to Lake Drūkšiai. The short-dashed line means the local railway route from the regional line to the INPP site. The scheme was made with Surfer software using data from geoportal.lt/map.

Lake Drūkšiai is the largest lake in Lithuania, located in the north eastern part of the country near the borders with Belarus and Latvia (Fig 1). Lake Drūkšiai itself has a length of 14.3 km, a width of 5.3 km, and a depth of up to 33 m. The surface area of the lake and the volume of its water are 49 km^2^ and 370 million m^3^, respectively. Lake Drūkšiai is a flow-through lake with 11 small streams flowing in and one stream flowing out, which has a water-level regulating dam. The lake is characterized by a relatively slow (3–4 y) water exchange rate and a high areal diversity of the bottom sediments.

The hydrographic network of Lake Drūkšiai catchment had undergone considerable changes in the 20th century. In about 1912, while constructing a water-mill, a canal was dug between Lake Drūkšiai and a small lake, located very close to Lake Drūkšiai. A runoff regulation sluice was installed after the construction of a 300 kW hydroelectric power plant (HEPP) downstream of a mentioned small lake in 1953. In the same year, one stream flowing from Lake Drūkšiai was blocked thereby increasing the catchment area by 25% (from 490 km^2^ to 613 km^2^). Artificial regulation of the water level reached 0.9 m (140.7–141.6 m a. s. l.), and the mean annual amplitude of water level fluctuation became 0.8 m or even 1.3 m. The HEPP was closed in 1982, shortly before the INPP was put into operation at the end of 1983.

For 26 years, Lake Drūkšiai served as a cooling basin for the INPP. Unit 1 came online in December 1983 and was shut down on December 31, 2004. Unit 2 was put into operation in August 1987 and shut-down on December 31, 2009. After the start of the INPP, the annual water level fluctuation amplitude decreased to 0.19–0.59 m (average value 0.4 m), as following the required regulations on lake water use and preservation, the annual lake water level fluctuation amplitude should not exceed 1.2 m to reduce the hazards of shore abrasion [10, 11].

Two (2019No2 and 2019No3) 41 cm length sediment cores were collected in September 2019 from a catamaran using a Kajak gravity corer with an acrylic sample tube. The cores were sectioned in 1 cm thick slices *in situ* using a piston rod immediately after sampling. The samples in containers were transported to the laboratory and stored at -40°C until analysis.

The sample site was chosen at the same place (within ~50–100 m) where the sediment core was taken in 2013 (55°38′49″ N; 26°35′07″ E) [5, 6]. This sampling station is located at the farthest distance from the INPP, in the area of the deepest depression (30–33 m) of Lake Drūkšiai (Fig 1). As an earlier study revealed [4], this area is also the main sedimentation area that has the highest inventory of ^137^Cs, ^60^Co and ^54^Mn, indicating that the redistribution and mechanical mixing of sediment layers are minimal. This area also has the lowest discharge of tributaries and outlets. The predominant fine silty mud in sediments [4] indicates primary production as the main source of sedimentary organic matter.

### Sample collection, preparation, and analysis

For radiocarbon measurements, sediment samples were prepared using the acid-base-acid (ABA) method to extract the alkali- soluble and -insoluble organic fractions as reported in the article [12].

We took into account Kleber and Lehmann’s argument [13] that alkaline extraction cannot separate humic substances from non-humic, so we decided not to use humus, humic acids, and other subcategories of humic substances, instead of using the terms as alkali -soluble and -insoluble sediment organic fractions.

The *Coregonus albula* and *Rutilus rutilus* scales, obtained from sets of 5 fish age of the third year (2+) for *Coregonus Albula*, and the fifth year (4+) for *Rutilus rutilus*, respectively, before radiocarbon dating were decalcified by immersing them in 1.2 N HCl for 2 min [14] (S1 Table). Measured samples of fish caught in 1983–1999 and 2005–2019 were taken from the collection kept at the Nature Research Center, Vilnius, Lithuania. All samples were stored at -40 °C until analysis. In conducting the research for this work, we did not work with living organisms. In this work data on the scales of *Coregonus albula* caught in 1983–1999 and 2005–2012 were taken from [5].

For the dating of the sediment layers, the measurements of ^210^Pb (at 46.5 keV), ^226^Ra (from ^214^Pb and ^214^Bi at 295, 352, and 609 keV), and ^137^Cs (at 661 keV) activity in lyophilized bottom sediment samples were performed by a gamma-ray spectrometer with HPGe GWL-series detector (resolution 2.25 keV at 1.33 MeV) using 3 cm^3^ geometry sample vials that fit the dimensions of well in an HPGe well-type detector as described in [4, 5]). Measurements were carried out in the layers of the core 2019 No3 (S1 Fig). The limit of detection for a counting time of 200,000 s was about 0.065 Bq for ^210^Pb, 0.021 Bq for ^226^Ra (from ^214^Pb) and 0.014 Bq for ^137^Cs, and the measurement errors did not exceed 15%, 20% and 8% for ^210^Pb, ^214^Pb, and ^137^Cs, respectively. Uncertainties (±2s) in the activity of radionuclides in the samples were evaluated using the GammaVision-32 software.

To reconstruct the chronology of recent sediments from the activity of ^210^Pb in lake sediments, the constant rate of the ^210^Pb supply (CRS) model [15–18] with some modifications [12, 19] was used (for more details see S1 File). As the total ^210^Pb activity in lake sediments has two components, supported activity resulting from the *in situ* decay of ^226^Ra in the sediments (in secular equilibrium with ^210^Pb) and unsupported ^210^Pb arising from atmospheric fallout and used for dating, the unsupported ^210^Pb activity was derived by subtracting ^214^Pb activity from the total ^210^Pb activity. The ^210^Pb excess was used for the sediment age calculation based on radioactive decay. The combination of ^210^Pb chronology with ^137^Cs activity peaks of 1963 and 1986 was used for validation of the age versus depth relationship for the lake sediment core (S3 Fig). Whereas the sediment core covers the period of 47 years (1973–2019), for analysis was used the data of the sediment core taken from a nearby station in 2013 and dated to 1950 CE (the lowest part) [5]

Pre-treated sediment samples of both cores, as well as fish scales, were graphitized using an Automated Graphitization Equipment AGE-3 (IonPlus AG). A single stage accelerator mass spectrometer (SSAMS, NEC, USA) was used for the ^14^C/^12^C ratio measurements of the graphitized sediment samples. The accuracy we obtained for the ^14^C/^12^C ratio was 0.3%. Phthalic acid anhydride was used for the estimation of the processed background and was determined to be 2.45×10^−3^ f_M_ (f_M_—the fraction of modern carbon). Alfa Aesar Black Carbon powder was used to test the SSAMS background, which was found to be 6.69×10^−4^ f_M_. IAEA-C3 (1.2941 f_M_) was used as reference material. The ^13^C /^12^C ratio value was used for the isotopic fractionation correction.

All results of radiocarbon dating measurements are reported in units of pMC (the percent of modern carbon):

$$pMC=f_{M}\times 100\%=\frac{A_{SN}}{A_{ON}}\times 100\text{\%,}$$ (1)

where A_SN_ is the specific activity of the sample A_S_, normalized to δ^13^C = −25‰; A_ON_ is the normalized activity of the standard [20, 21]. However, ^14^C specific activity in Bq/kg C and the percent of modern carbon (pMC) are related to 2.26 Bq/kg C: 1 pMC [20], so the ^14^C measurement results were reported as the ^14^C specific activity in pMC.

The radiocarbon reservoir age (RRA) calculations were used to evaluate ^14^C liquid releases from the INPP. The radiocarbon reservoir age values in organic sediment fractions were calculated according to the following equation [22]:

$$RRA=8033\times \text{ln}\left(\frac{pMC_{T}}{pMC_{A}}\right)\text{,}$$ (2)

where pMC_A_ and pMC_T_ are ^14^C specific activity values in aquatic and terrestrial samples of the same period, respectively. As pMC_T_, we used ^14^C specific activity measurements in P. *Sylvestris* tree ring samples. Samples for the period of 1957–1983 were taken from the background location (rural area 165 km away from the INPP, (54°46’92" N, 24°77’81" E)), for the period of 1984–2019 were taken the tree-rings of the tree growing 6.6 km away from the INPP [8].

The hypothetical radiocarbon specific activity values in alkali -soluble and -insoluble sediment organic fractions that would have occurred without any impact of the INPP were calculated using the results of our previous measurements in a sediment core taken in 2013 (S4 and S5 Figs), where it was determined [6] that the RRA values of both fractions returned their former pre-bomb values at the beginning of the INPP operation, approximately in 1986. Thus, hypothetical ^14^C specific activity values for the organic fractions of both cores for the period of the INPP operation were determined from Eq 2, using a constant RRA values of 1986. The contribution of each fraction to the total ^14^C activity accumulated in the sediments was determined from the carbon accumulation rates in both fractions.

C/N values in bulk sediments (S6 Fig), as well as organic carbon concentrations in both sediment fractions to estimate the carbon accumulation rate in fractions (S3 Table) were measured with an elemental analyser (Thermo Flash EA 1112). The long-term standard measurements were performed with a precision of <1.8% for C and <1.5% for N.

## Results and discussion

**Fig 2:**
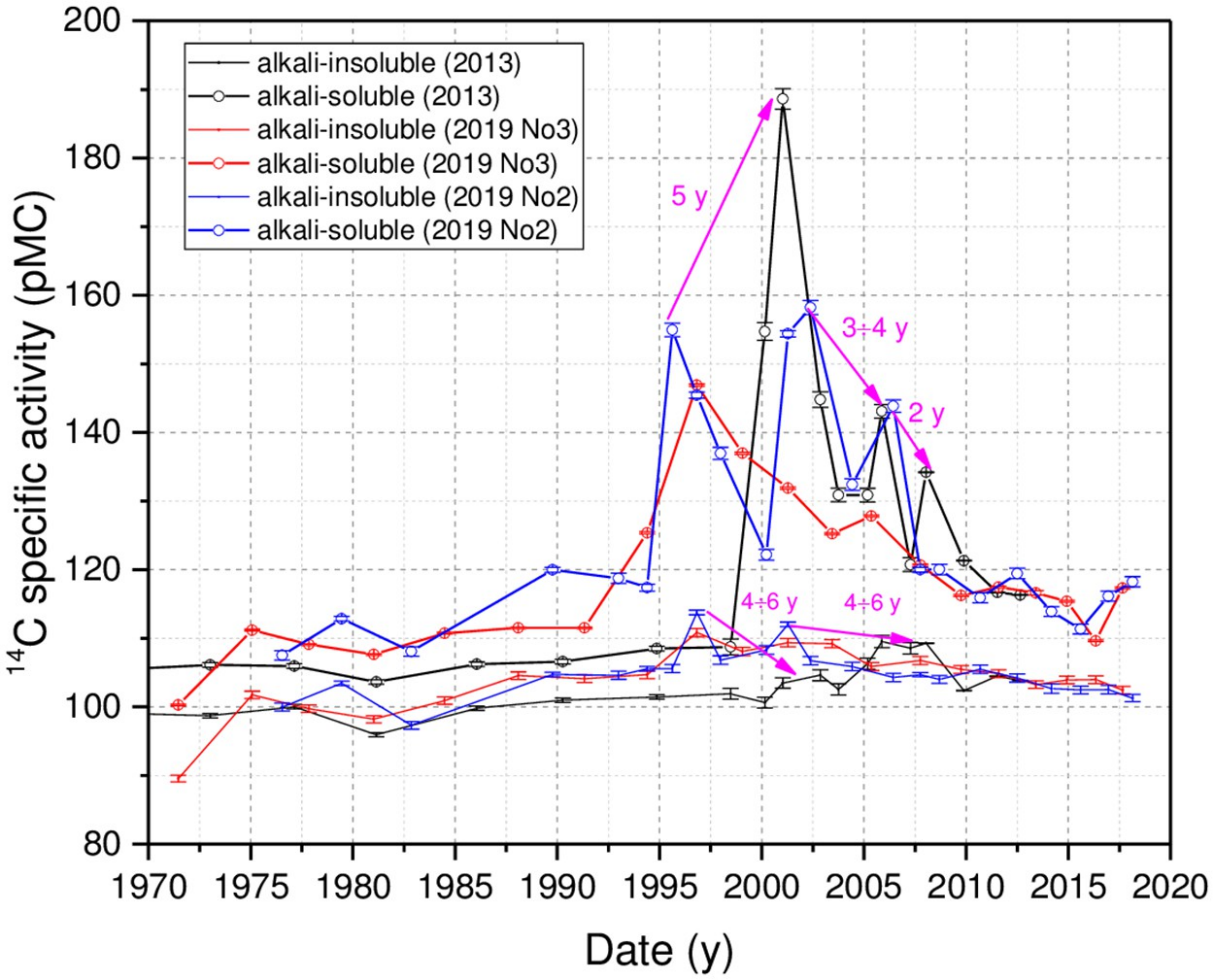
^14^C specific activity in the alkali-soluble and alkali-insoluble sediment fractions. Data of the sediment core taken in 2013 are from [5].

The ^14^C content in two organic sediment fractions from all three cores for the period of 1971-2018/2019 are shown in Fig 2. During the periods 2000 to 2002 and 2004–2008, a sharp increase (up to 80 pMC and 40 pMC, respectively) of ^14^C specific activity in the alkali-soluble organic fraction, as well as up to a few pMC increase in the alkali -insoluble fraction of the core taken in 2013, repeated in the cores of 2019, but a few years earlier (1995–2000, 2001–2003, 2004–2007 as well as 1995–2003, 2004–2007 for cores 2019 No2 and 2019 No3, respectively). No sharp increase in ^14^C specific activity was observed in core 2019No3 during the period 2001–2003. The ^210^Pb and ^137^ Cs dating were performed in the core 2019 No3 (S1 File).

**Fig 3:**
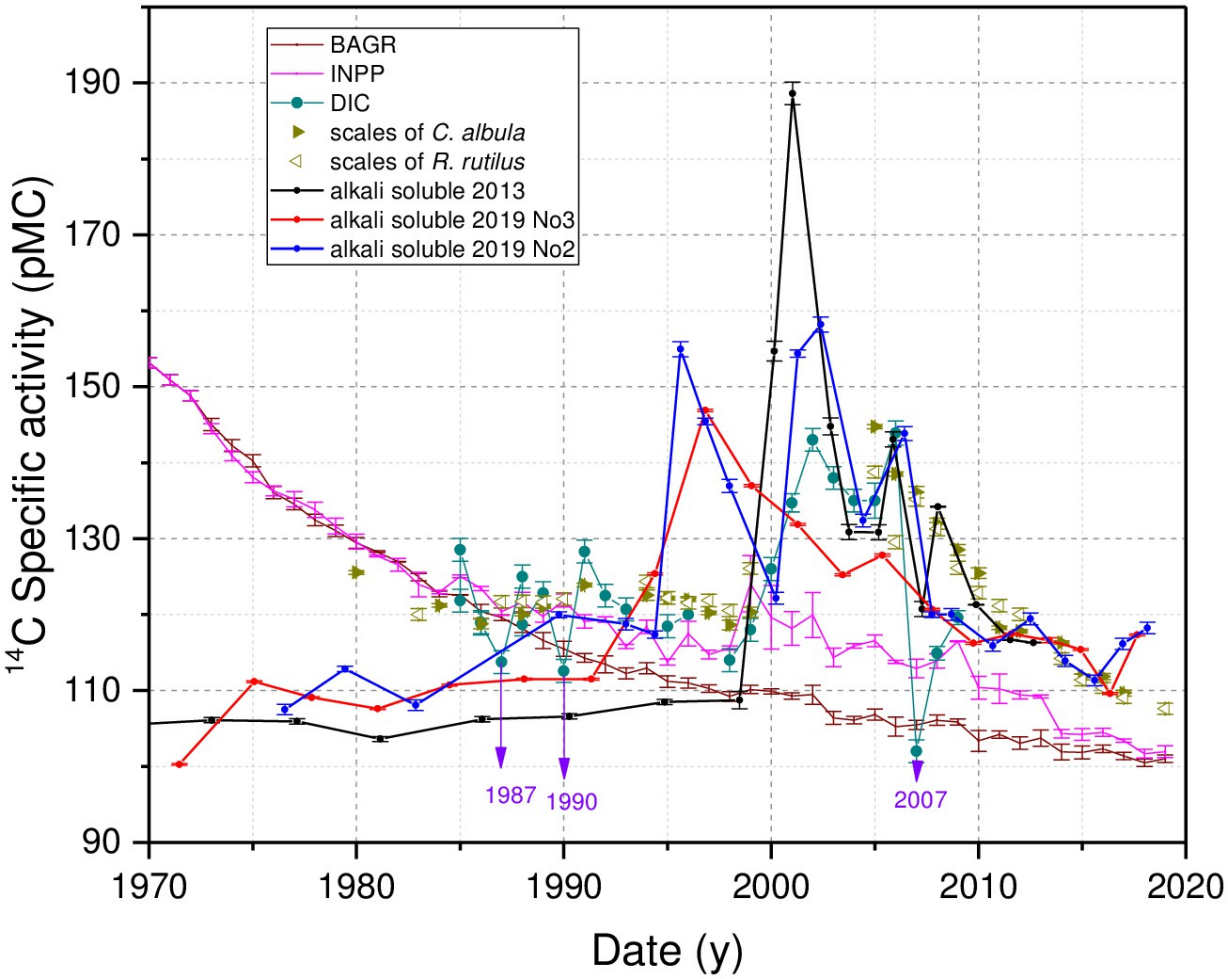
^14^C specific activity in the alkali-soluble sediment fractions of all three cores, in DIC (data from [23]) during the period of INPP operation, in the scales of *C*. *albula* (data of 1980–2012 taken from [5]) and *R*. *rutilus*, as well as in the *P*. *sylvestris* tree rings from the INPP area (INPP) and the background area (BAGR) located 165 km southwest of the INPP (data taken from [8]).

The discrepancies in the periods of sudden increase in ^14^C specific activity in the alkali -soluble organic fraction in the cores taken in 2013 and 2019 could be related to dating inaccuracies. This can be justified by the following observations. First, there could not be an increased deposition of terrestrial origin organic matter from the ‘bomb peak’ period at the sampling site of 2019, at least from 1995 to 2000. The lake water level has been kept constant since 1983; SMAR (sediment mass accumulation rates) data for the core do not show any increase in sedimentation rates (S2 Fig), and the C/N measurement data (S6 Fig) did not show any change in the concentration of terrestrial origin organic matter during that period either. Second, the DIC samples measurements taken from [23] do not show an increase in ^14^C-specific activity in 1995–2000. Unfortunately, the DIC sampling site was located close to the Gulbinėlė streamlet, connecting small Lake Skripkai to Lake Drūkšiai (JM 6 (Table 1)). Measurements of water samples collected in Lake Skripkai from 1989 to 2001 (S2 Table) showed that the values of ^14^C specific activity in DIC varied between 67–97 pMC (only in May 1992 and 1995 the values exceeded 100 pMC). Thus, the inflowing water from Lake Skripkai depleted in ^14^C could affect (depending on the prevailing wind direction during sampling) the radiocarbon specific activity values in the DIC samples, as can be seen in samples from 2007 and possibly from 1987 and 1990 (Fig 3). The water samples for the DIC measurement in Lake Drūkšiai were collected only once or twice a year. There are no data on variations in DIC concentration and isotopic composition related to ongoing processes in the lake (as e.g., the peak of phytoplankton blooming, wthen daily pH values in boreal lakes can change up to 1 [24]).

**Table 1:** ^14^C specific activity (pMC) measured in aquatic plants of Lake Drūkšiai collected during 1996–2019.

Station	Species	^14^C_plants_, pMC	^14^C_bagr_, pMC
1996			
JM1 (sediment core sampling station)	*Myriophyllum spicatum*	131.9±6.0	111.00±0.67
JM 4 (near Outlet)	*Clodophora glomerata*	120.7±5.7	111.00±0.67
JM 4 (near Outlet)	*Myrophillum spicatum*	121.4±2.7	111.00±0.67
2002-07-03			
M1	*Ceratophyllum demersum*	131±1	109.45±1.26
M2	*Cladophora glomerata*	145±2	109.45±1.26
M3(Outlet)	*Chara vulgaris*	153±2	109.45±1.26
M4	*Myriophyllum*	147±2	109.45±1.26
M5(close to IRD-3)	*Chara vulgaris*	141±2	109.45±1.26
M6-1 (close to the Inlet)	*Potamogeton*	155±2	109.45±1.26
M6-2 (close to the Inlet)	*Ceratophyllum demersum*	150±2	109.45±1.26
2007-06-07			
JM 1 (sediment core sampling station)	*Myriophyllum*	119.3±1.9	105.48±0.60
JM 1(sediment core sampling station)	*Cladophora glomerata*	111.1±0.9	105.48±0.60
JM 4 (near Outlet)	*Ceratophyllum demersum*	111.6±1.5	105.48±0.60
JM 6	*Ceratophyllum demersum*	106.3±1.5	105.48±0.60
JM 7 (near IRD-1,2)	*Ceratophyllum demersum*	172.3±2.8	105.48±0.60
JM IRD_1,2	*Ceratophyllum demersum*	262.6±2	105.48±0.60
JM Outlet	*Myriophyllum*	108.8±1.6	105.48±0.60
2008-08-27			
JM 1 (sediment core sampling station)	*Ceratophyllum demersum*	118.8±1.0	106.10± 0.68
JM 4 (near Outlet)	*Myriophyllum spicatum*	125.9±0.7	106.10± 0.68
JM 7 (near IRD-1,2)	*Ceratophyllum demersum*	123.4±0.9	106.10± 0.68
JM IRD_1,2	*Ceratophyllum demersum*	172.9±7.7	106.10± 0.68
JM (Outlet)	*Myriophyllum spicatum*	127.7±0.6	106.10± 0.68
2009-08-21			
JM 1 (sediment core sampling station	*Cladophora glomerata*.	129.5±1.2	105.86 ±0.39
JM 1(sediment core sampling station)	*Ceratophyllum demersum*	125.0±1.2	105.86 ±0.39
JM 4 (near Outlet)	*Myriophyllum spicatum*	122.7±2.7	105.86 ±0.39
JM 7 (near IRD-1,2)	*Potamogeton sp*.	135.7±1.0	105.86 ±0.39
JM IRD_1,2 kanalo pradžia	*Myriophyllum spicatum*	233.4±1.0	105.86 ±0.39
JM IRD_1,2 kanalo vidurys	*Myriophyllum spicatum*	387.2±2.6	105.86 ±0.39
JM IRD_1,2 kanalo pabaiga	*Ceratophyllum demersum*	233.1±2.9	105.86 ±0.39
JM Outlet (Outlet)	*švendrų šaknys*	147.0±0.9	105.86 ±0.39
2017-08-13			
JM 1 (sediment core sampling station	*Elodea canadensis*	110.4±2.9	101.38±0.49
JM 4 (near Outlet)	*Ceratophyllum demersum*	106.4±1.0	101.38±0.49
JM 6	*Potamogeton*	107.0±0.7	101.38±0.49
2019-09-13			
1-1(close to JM 1)	*Myriophyllum spicatum*	107.64± 0.38	101.02±0.50
1-1(close to JM 1)	*Ceratophyllum demersum*	107.95± 0.38	101.02±0.50
1-1(close to JM 1)	*Potamogeton*	107.16±0.45	101.02±0.50

^14^Cbagr—the ^14^C specific activity in *P*. *sylvestris* tree rings from the background area. Data was taken from [8]

IRD-1,2,3—industrial discharge and rainwater drainage (IRD) channels 1, 2, 3.

Inlet—cooling water inlet channel

Outlet—heated water outlet channel.

JM 1 the deepest depressions of the lake, sediment core sampling site

JM 4 was located near discharges from Outlet

JM 6 was located near the Gulbinėlė streamlet connecting Lake Drūkšiai with Lake Skripkai.

JM 7was located near discharges from IRD-1,2

M3 Outlet channel

M5 was located near discharges from IRD-3

M6 was located close to the Inlet channel

Data for 2007–2008 (stations were marked with JM) was taken from [23]. Data for 2002 was taken from [32].

However, measurements in scales of *C*. *albula* caught between 1995 and 2000 (Fig 3) also showed no increase in ^14^C specific activity by 40–43 pMC. *C*. *albula* (vendace) feeds exclusively on zooplankton [25–27]. Thus, bioaccumulation of ^14^C in this fish through a relatively short food chain: *dissolved inorganic carbon (DIC) → aquatic primary producers → zooplankton → C*. *albula* is expected. As fractionation of the ^14^C isotope during trophic transfer is already included in ^14^C measurement values by the δ^13^C correction [20], *C*. *albula* scales reflect averaged ^14^C specific activity values in the DIC of lake water over their lifetime of ~2 y, (S1 Table). The ^14^C measurement values in scales of *R*. *rutilus* (common roach) were very similar to *C*. *Albula* during 1995–1998. ^14^C specific activity values in both fish varied within 1.8 pMC (Fig 3, S1 Table) even though *C*. *Albula* reflect ^14^C specific activity values in the DIC but averaged over ~ 4 y. This fish was about the same age for the ontogenetic shift (of 3–4 years) from a zooplanktivore into a benthic omnivore, then the diet mainly consisting of molluscs, especially dreissenids [28–31], thus, the higher ^14^C value (of 126.08±0.75 pMC) in scales of fish caught in 1999 could be related with differences in food source. Unfortunately, there are no fish samples from the period of 2000–2004 in order to verify the differences in the specific radiocarbon activity of both fish species and to evaluate/assess the influence of benthic food sources on the ^14^C concentration in fish tissues.

Significant changes in the ^14^C distribution between the two interacting sediment organic fractions are seen during the periods of 2000 to 2002 and 2004–2008 as well as the periods of 1995–2003, 2004–2006 in the cores taken in 2013 and 2019, respectively, which indicate the possibility that another form of radiocarbon, rather than DIC, may have been discharged to the lake.

These changes in ^14^C distributions between sediment organic fractions could not be related with terrestrial origin organic matter. As mentioned before, water level in the lake was kept constant since 1983 (the values of C/N measurements did not exceed 10 (S6 Fig) indicating that the majority of sedimentary organic matter was of autochthonous origin [33]).

Previous research showed that low molecular weight (<3.5 Da) xenobiotics and their metabolites can be easily taken up and accumulated by freshwater plants and organisms [34–37]. The ^14^C specific activity in the tissues of the plant should reflect the average ^14^C specific activity values in DIC and in dissolved organic carbon (DOC) that could be possibly accumulated during its growing season. However, ^14^C enriched water-soluble organic compounds are not expected to be released during routine operation processes of RBMK-type reactors. As can be seen in Table 1, even ^14^C specific activity measurements in aquatic plants indicate that the concentrations of ^14^C in water discharged through the heated water outlet channel (the station JM Outlet) are certainly not the highest compared to the concentrations measured in plants from other parts of the lake. The highest radiocarbon specific activity values had the plants collected nearly or in the industrial discharge and rainwater drainage (IRD) channels 1, 2. Thus, these channels could have released dissolved organic compounds that might have been used in processes related to the maintenance and decontamination of the technological circuits of the plant. Unfortunately, sediment organic compounds are affected by continuously ongoing decomposition processes of larger molecules as well as the incorporation of smaller size compounds into larger aggregates; thus, additional analyses such as NMR, Py-GC-MS, and FTICR-MS related to the identification of changes in individual chemical groups would not necessarily reveal the primary polluting compounds. The possible decomposition of these compounds in the water column should also be taken into account [38].

**Fig 4:**
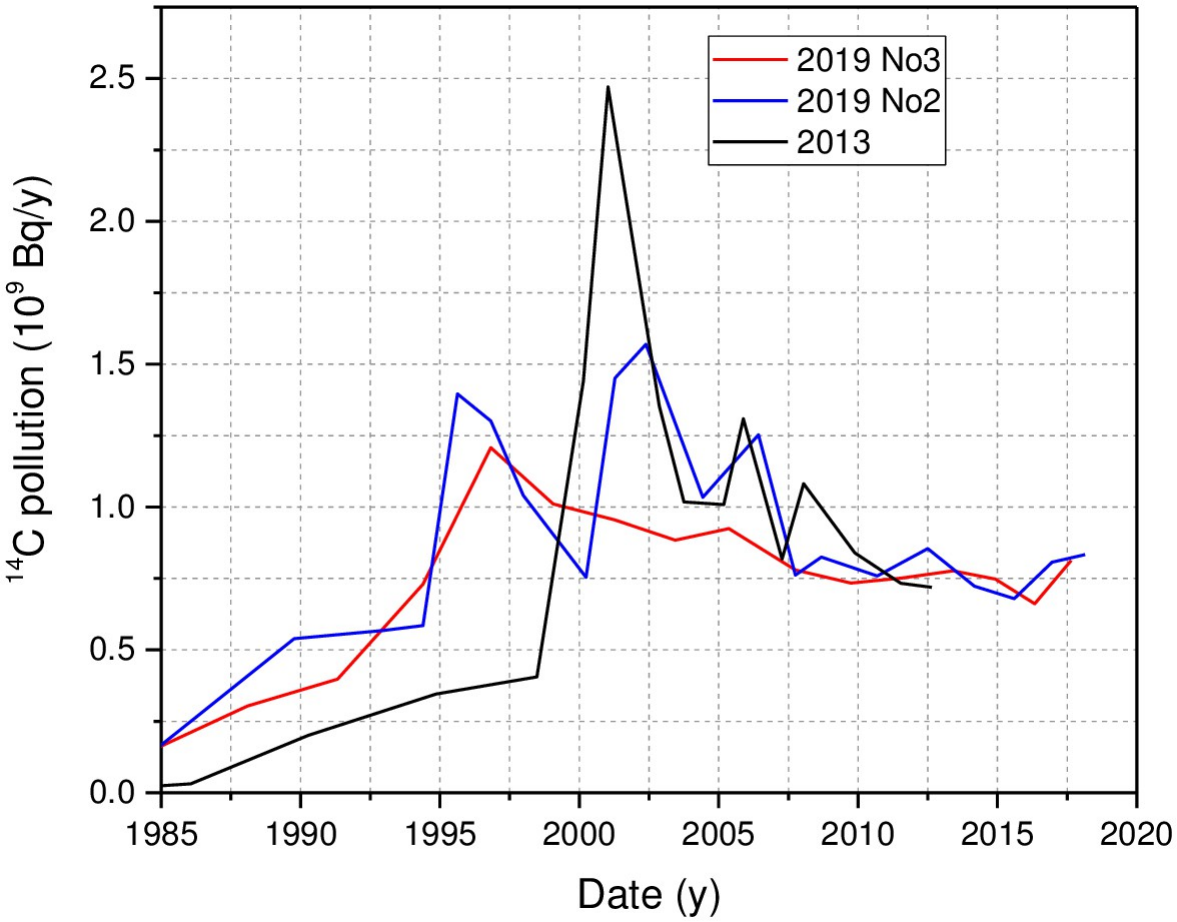
Evaluated INPP impact in terms of released ^14^C activity. Data for core 2013 was taken from [5, 6]. Calculations were performed using estimated radiocarbon specific activity that would have occurred without any INPP impact (S5 Fig).

As can be seen in Fig 4, until the period of increased ^14^C pollution in 2000 and 1995 as revealed by the cores sampled in 2013 [6] and 2019, respectively, up to 0.37 ÷0.6 × 10^9^ Bq/y of ^14^C was introduced into Lake Drūkšiai. During the periods 1995–2000, 2001–2003, 2004–2007, core 2019No2 showed a ^14^C release rate from the INPP to water up to 1.31×10^9^ Bq/y, 1.57×10^9^ Bq/y, and 1.43×10^9^ Bq/y, respectively, while ^14^C releases seen in the organic fractions of the core 2019No3 were lower up to 1.24×10^9^ Bq/y, and 0.94×10^9^ Bq/y for the periods 1995–2003, 2004–2007, respectively. Radiocarbon specific activity measurements in the organic fractions of the core taken in 2013 showed higher ^14^C discharges to the lake (up to 2.3 × 10^9^ Bq/y).

After the final shutdown of the INPP at the end of 2009, the ^14^C release rate to the lake (data of the cores taken in 2019) remains quite constant at 0.75±0.2 10^9^ Bq/y for all nine years. After 2016, an increase in ^14^C specific activity by 9 pMC (from 110 ±1 pMC to 119±0.6 pMC) is seen (Fig 3 and S5 Fig) in the alkali -soluble fraction of both cores taken in 2019 (but not in the alkali -insoluble fractions). This spill of compounds enriched in ^14^C was not measured on scales of R. *rutilus* that reflected DIC (and partly DOC) for a period of 4 years. The reason might be a too low concentration of compounds in the water column to change ^14^C specific activity on fish scales. In addition, we cannot exclude the possibility of the appearance of other types of compounds with different accumulation abilities in fish tissues.

Differences in the estimates of liquid ^14^C routine releases from the INPP using radiocarbon measurements in the sediment cores may be related to the following reasons. First, in the calculations, we used the RRA values of the pre-bomb period, before 1952 ±5–9 y (dating error) (data from the core taken in 2013 [5]). These values were possibly affected by the rise in water level in 1953, due to the construction of the HEPP on the Prorva River (Belarus) that caused a 25% increase in the catchment area [10]. This increase in the catchment area is seen in the SMAR data (S2 Fig) and definitely could impact carbon cycling in the lake leading to changes in the RRA after the 1953s. Artificial regulation of the water level by the HEPP caused the mean annual amplitude of water level fluctuations to be up to 1.3 m until the beginning of the INPP operation in 1983. It is impossible to evaluate how these water level fluctuations affected the RRA during this period (1953–1983), as after the 1953s, intensification of nuclear weapons testing caused an increase in atmospheric radiocarbon concentration. Second, it was not possible to assess the impact of eutrophication on the RRA. In the 1960s, intensive agriculture development in the Soviet Union began, followed by the input of mineral fertilizers into rivers and lakes (peaked in 1989–1990), which led to an intensification of eutrophication. Lake Drūkšiai from the (oligo) mesotrophic became almost eutrophic due to the effluents that carried nitrogen and phosphorus compounds not only from mineral fertilizers but also from the wastewater of the Visaginas town, established in 1975. Third, it is not possible to exclude the impact of the INPP activities not related to ^14^C pollution on the carbon cycle of the lake ecosystem leading to changes in the RRA. After the start of the INPP, the annual water level fluctuation amplitude was maintained at 0.4 m to reduce the hazards of shore abrasion due to safety reasons [39]. This may have caused changes in the carbon cycle. However, significant changes in the cycle of organic and inorganic matter in the lake should have been associated with changes in the thermal regime [40]. An increase in temperature caused changes in the carbon cycle of the lake due to the impact on the lake’s flora and fauna [41–43], water mixing, as well as organic and inorganic matter production, decomposition, utilization, sedimentation, export, exchange rates of CO_2_, CH_4_ (as well as other gases related to photosynthesis and processes of decomposition) with the atmosphere.

Fourth, huge water masses involved in the cooling cycle of INPP different operating modes induced changes not only in the sedimentation rate, but also in the composition of the upper layers of sediments in most of the lake area [44]. All investigated sediment cores were taken within 50–100 meters of each other (since cores of 2019 were taken from a catamaran, the distance between their sampling sites could be up to 20 m). It is well known that the radiocarbon distribution in dissolved and particular organic matter, as well as in DIC varies at different locations in the lake [45, 46]. This depends on the air-water CO_2_ exchange rate related to organic matter production and decomposition, organic and inorganic matter import/export, water residence time [45–48] at each site. However, the influence of water mass circulation associated with INPP operation processes on the mixing, resuspension, and remobilization of sediments was insignificant only in the area of the deepest basin of the lake (the place where sediments were sampled). Therefore, our research reveals the situation in this particular area, but not in the whole lake.

## Conclusions

Comparison of the vertical distribution of radiocarbon in the three undisturbed bottom sediment cores taken within an area of 50–100 m size in the deepest depression of Lake Drūkšiai in 2013 and 2019 revealed some differences in ^14^C activity concentration profiles. Typically, radiocarbon distribution studies are not performed on multiple sediment cores from the same lake site, as it is simply not expected that the carbon cycle at the closely located sites would be affected by different factors. However, our study has shown that cores taken at the same lake site (both cores of 2019 were taken at a distance of 10–20 m from each other) can have different ^14^C distributions in the corresponding layers of the core with a difference of up to 30 pMC (or the corresponding ^14^C release rate of 0.7 GBq/y).

Based on the cores taken in 2019, episodes of increased release of ^14^C began in 1995 and lasted until 2006. While episodes of contamination were sharper, more pronounced, and corresponded to a somewhat later period of 1999–2008 based on a core taken in 2013.

According to a core taken in 2013, the ^14^C release rate increased to 2.48 GBq/y during the 1999–2003 pollution episode, while during the 2004–2008 episode, the ^14^C release rate did not exceed 1.3 GBq/y. Data attributed to cores 2019 No2 and 2019 No3 showed that during the 1995–2000 episode, ^14^C release rate increased from 0.6 GBq/y to 1.25 GBq/y and 1.2 GBq/y, respectively. During a 2001–2003 episode, an increase in the ^14^C release rate to 1.57 GBq/y was derived from the data of core 2019 No2, and a twice lower value was obtained from the data of core 2019 No3. Both cores sampled in 2019 showed permanent ^14^C release rate of 0.76±0.06 GBq/y since 2006 including all eight years after the closure of the INPP.

Overall, the ^14^C release rate estimated from radiocarbon measurements in all three cores taken at different times did not differ by more than 1.3 GBq/y. It should be noted that the ‘pre-bomb’ RRA value was used in the release rate estimation calculations to determine the radiocarbon specific activity values that would have occurred without any INPP impact. However, the carbon cycle of the lake was influenced by other factors associated with anthropogenic activity not related to ^14^C pollution, including hydrological changes in the lake, an increase in the temperature that affected all processes in the lake’s ecosystem. All of such factors could lead to variation of the estimated liquid radiocarbon discharges from INPP.

## Supporting information

S1 File Dating lake sediments by ^210^Pb and ^137^Cs and CRS sedimentation rates. (PDF)

S1 Table Biological parameters of the planktivorous vendace (*Coregonus albula*) and common roach (*Rutilus rutilus*) samples from Lake Drūkšiai and ^14^C activity data in fish scales. Data is shown as mean ± standard deviation whenever possible.
(PDF)

S2 Table ^14^C specific activity measurements in DIC samples, taken from Lake Skripkai during 1989–2001. (PDF)

S3 Table ^14^C specific activity measurements in organic fractions of sediment columns 2019 No2 and 2019 No3, carbon accumulation rates in sediment organic fractions, and extent of ^14^C in sediments, estimated from measurements in both columns. (PDF)

S1 Fig Lake Drūkšiai sediment records (core 2019 No3): A) ^137^Cs and ^210^ Pb profiles; error bars represent uncertainties based on the propagation of 2 σ counting errors; B) model-determined sediment ages from the ^210^Pb profile. (TIF)

S2 Fig Sediment mass accumulation rates (SMAR) based on ^210^Pb chronology (core 2019 No3). (TIF)

S3 Fig ^137^Cs profiles for two sediment cores (2013 and 2019 No3). (TIF)

S4 Fig ^14^C specific activity in the alkali-soluble and alkali-insoluble sediment fractions of the sediment core, taken in 2013 (data from [2] were used), calculated RRA data on both fractions, and radiocarbon specific activity that would have occurred without any INPP impact. (TIF)

S5 Fig Measured values of ^14^C specific activity in the alkali-soluble and alkali-insoluble sediment fractions in both cores of 2019 and calculated radiocarbon specific activity that would have occurred without any impact of the INPP. (TIF)

S6 Fig C/N ratio values in sedimentary organic matter of the column 2019No3. (TIF)
