# Supplementary material for: What cooling pond sediments can reveal about 14C in nuclear power plant liquid effluents: Case study Lake Drūkšiai, Ignalina nuclear power plant cooling pond
Source: PLoS One. 2023 Oct 20;18(10):e0285531. doi: 10.1371/journal.pone.0285531 (PMC10588893; doi:10.1371/journal.pone.0285531)
Supplement: S1 File — (PDF) [file pone.0285531.s001.pdf]

Dating lake sediments by ^{210}Pb and ^{137}Cs and CRS sedimentation rates

The data on the activity concentration of ^{210}Pb (total), ^{214}Pb and ^{137}Cs in the sediment core 2019 No3 versus depth in 1 cm resolution to 41 cm depth are given in S1A Fig.

The specific activity of the total ^{210}Pb exponentially decreased from 460–490 Bq/kg in the 0–3 cm depth interval of the sediment core to 130–136 Bq/kg at a depth of 40–41 cm, however, with positive and negative ^{210}Pb deviations from exponential function. These small changes could be influenced by many factors, including the ^{210}Pb atmospheric flux and the rate of its transport from the catchment; the water residence time; the fraction of radionuclides attached to settling particles and mean particle settling velocity; various post-depositional transport processes [1].

Based on the conventional statistics (^{214}Pb average value in sediment core plus 2 standard deviations), the maximal level of ^{214}Pb in sediments from decaying of ^{226}Ra *in situ* was evaluated to be 86 Bq/kg. In the studied core, at a depth of 41 cm, there is still an excess of ^{210}Pb, $^{210}Pb_{ex}$, relative to ^{214}Pb up to 40-45 Bq/kg. The ^{210}Pb excess was used for the sediment age calculation based on radioactive decay using the Constant rate of supply (CRS) model (model-determined sediment ages are given in S1B Fig.). The profile of $^{210}Pb_{ex}$ in the sediment core to a depth of 41 cm tended to decrease exponentially with the mass depth, the product of the wet thickness of the sediment slice and the dry bulk density, d_m ($^{210}Pb_{ex}=358\times\exp(-0.326\times d_m)$; $R^2=0.93$). This evidenced a rather constant $^{210}Pb_{ex}$ flux and steady-state sedimentation with an average sediment mass accumulation rate (SMAR) of 0.10 g/cm^2/y. This average SMAR value was used to calculate the sediment age and partial SMAR values for the studied core up to the depth of 41 cm (S2 Fig).

The sediment age of the lowest part of the core can be dated to 1971.4±2.2 CE. The partial SMAR values for the past 50 years have been in the range of 0.075 ± 0.025 to 0.128 ± 0.028 g/cm^2/y with an average SMAR value of 0.13 g/cm^2/y. The average of 2σ uncertainties for the SMAR was 23%. The average SMAR value corresponded to the linear sedimentation of wet matter equal to 0.96 cm/y.

The ^{210}Pb chronology of our core was compared with that of longer core (60 cm) dated to 1950 CE (the lowest part) and taken from a nearby station in 2013 [2]. The combined data on ^{137}Cs from both cores plotted versus ^{210}Pb-derived time evidenced synchronous changes of ^{137}Cs (S3 Fig).

Further, the data on ^{137}Cs fallouts from the atmosphere due to the Chernobyl NPP accident and nuclear weapons testing in the atmosphere, as independent chronostratigraphic markers well confirmed chronology based on ^{210}Pb. For longer core of 2013 two peaks of ^{137}Cs at the sediment depths of 49 and 33 cm corresponded to the ^{210}Pb dates of ~1963.2 ± 2.2 and ~1986.1 ± 1.0 CE. For shorter core of 2019, one peak dated to 1986.1± 0.9 CE was clearly traced at a depth of 32.5 cm and the beginning of second peak was also evident.

References

1. Appleby PG. Three decades of dating recent sediments by fallout radionuclides: a review. 2008;18: 83–93. doi:10.1177/0959683607085598
2. Barisevičiūtė R, Maceika E, Ežerinskis Ž, Šapolaitė J, Butkus L, Mažeika J, et al. Distribution of radiocarbon in sediments of the cooling pond of RBMK type ignalina nuclear power plant in Lithuania. PLoS One. 2020;15(8): e0237605. doi:10.1371/journal.pone.0237605
