# Supplementary material for: What cooling pond sediments can reveal about 14C in nuclear power plant liquid effluents: Case study Lake Drūkšiai, Ignalina nuclear power plant cooling pond
Source: PLoS One. 2023 Oct 20;18(10):e0285531. doi: 10.1371/journal.pone.0285531 (PMC10588893; doi:10.1371/journal.pone.0285531)
Supplement: S1 Table — Data is shown as mean ± standard deviation whenever possible. (PDF) [file pone.0285531.s002.pdf]

S1 Table. Biological parameters of the planktivorous vendace (*Coregonus albula*) and Common roach (*Rutilus rutilus*) samples from Lake Drūkšiai and ^{14}C activity data in fish scales. Data is shown as mean ± standard deviation whenever possible.

Year	***Coregonus albula***				***Rutilus rutilus***			
	n	TL (cm)	Age (y)	^{14}C (pMC)	n	TL (cm)	Age (y)	^{14}C (pMC)
1980	*5*	*19.8 ± 0.5*	*2+*	*125.55±0.25*				
1983					5	13.0 ± 3.6	4+	120.02±0.78
1984	*5*	*19.6 ± 1.2*	*2+*	*121.20±0.24*				
1986	*5*	*19.1 ± 0.9*	*2+*	*118.80±0.65*				
1987					5	16.6 ± 1.7	4+	121.69±0.82
1988	*5*	*18.8 ± 0.7*	*2+*	*120.05±0.24*	5	16.2 ± 1.6	4+	121.74±0.82
1989	*5*	*18.4 ± 0.9*	*2+*	*121.05±0.24*	5	15.2 ± 0.8	4+	121.64±0.82
1990					5	12.2 ± 3.1	4+	122.05±0.83
1991	*5*	*18.1 ± 0.8*	*2+*	*123.90±0.24*				
1994	*5*	*17.6 ± 0.5*	*2+*	*122.55±0.68*	5	15.1 ± 1.6	4+	124.36±0.84
1995	*5*	*17.9 ± 0.6*	*2+*	*122.16±0.24*	5	15.3 ± 1.2	4+	122.20±0.83
1996	*5*	*18.5 ± 0.8*	*2+*	*121.95±0.24*	5	14.6 ± 1.2	4+	121.54±0.82
1997	*5*	*17.6 ± 0.6*	*2+*	*120.21±0.24*	5	13.6 ± 0.1	4+	121.86±0.82
1998	*5*	*17.7 ± 0.6*	*2+*	*118.63±0.65*	5	13.5 ± 0.3	4+	120.53±0.82
1999	*5*	*18.8 ± 0.7*	*2+*	*120.32±0.66*	5	14.4 ± 0.3	4+	126.08±0.75
2005	*5*	*18.9 ± 1.0*	*2+*	*144.74±0.28*	5	13.2 ± 1.3	4+	138.78±0.79
2006	*5*	*19.3 ± 0.8*	*2+*	*138.53±0.27*	5	13.3 ± 1.5	4+	129.53±0.88
2007	*5*	*18.4 ± 1.2*	*2+*	*136.11±0.75*	5	13.4 ± 0.5	4+	135.17±0.91
2008	*5*	*17.3 ± 0.3*	*2+*	*131.98±0.73*	5	13.1 ± 1.1	4+	131.27±0.89
2009	*5*	*18.6 ± 1.0*	*2+*	*128.52±0.71*	5	14.1 ± 1.2	4+	126.15±0.85
2010	*5*	*18.6 ± 0.7*	*2+*	*125.46±0.69*	5	13.2 ± 1.0	4+	122.88±0.83
2011	*2*	*19.7 ± 0.9*	*2+*	*118.29±0.23*	5	15.4 ± 3.1	4+	121.17±0.82
2012	*5*	*20.0 ± 0.7*	*2+*	*117.82±0.23*	5	14.9 ± 4.6	4+	120.05±0.81
2014	5	19.6 ± 0.7	2+	116.31±0.23	5	15.9 ± 3.5	4+	114.20±0.77
2015	5	19.7 ± 0.7	2+	111.80±0.40	5	13.0 ± 0.8	4+	111.39±0.75
2016	5	19.8 ± 0.7	2+	111.85±0.40	5	12.9 ± 0.9	4+	110.35±0.71
2017	5	19.0 ± 0.1	2+	109.66±0.40	5	14.5 ± 3.1	4+	109.04±0.74
2019				107.24±0.40	5	15.5 ± 3.1	4+	107.60±0.73

This is S1 Table legend.

Data on *Coregonus albula* scales caught in 1980-1999 and 2005-2012 were taken from [2].
