# Supplementary material for: What cooling pond sediments can reveal about 14C in nuclear power plant liquid effluents: Case study Lake Drūkšiai, Ignalina nuclear power plant cooling pond
Source: PLoS One. 2023 Oct 20;18(10):e0285531. doi: 10.1371/journal.pone.0285531 (PMC10588893; doi:10.1371/journal.pone.0285531)
Supplement: S2 Table — (PDF) [file pone.0285531.s003.pdf]

S2 Table. ^{14}C specific activity measurements in DIC samples, taken from Lake Skripkai during 1989-2001.

**Year**	**^{14}C (±2 pMC)**
1989-06	85.39
1989-09-09	82.95
1990-02	121.7
1992-06-13	75.82
1993-09	67.45
1994-06	97.31
1995-05-27	118.04
1996	74.6
1998	85.23
1999	92.58
2000	88.2
2001	91.7

This is S2 Table legend.
