# Supplementary material for: What cooling pond sediments can reveal about 14C in nuclear power plant liquid effluents: Case study Lake Drūkšiai, Ignalina nuclear power plant cooling pond
Source: PLoS One. 2023 Oct 20;18(10):e0285531. doi: 10.1371/journal.pone.0285531 (PMC10588893; doi:10.1371/journal.pone.0285531)
Supplement: S3 Table — (PDF) [file pone.0285531.s004.pdf]

S3 Table. ^{14}C specific activity measurements in organic fractions of sediment columns 2019 No2 and 2019 No3, carbon accumulation rates in sediment organic fractions, and extent of ^{14}C in sediments, estimated from measurements in both columns.

Year	$^{14}C_{alkali\_insoluble}$, pMC	$^{14}C_{alkali\_soluble}$, pMC	$C_{alkali\_insoluble}$, g cm^{-2} y^{-1}	$C_{alkali\_soluble}$, g cm^{-2} y^{-1}	^{14}C pollution ×10^9, Bq/y
**2019 No2**					
2018	101.27±0.53	118.24±0.75	0.00146	0.00424	0.83364
2017	102.52±0.60	116.14±0.74	0.00156	0.0041	0.80676
2016	102.47±0.57	111.34±0.71	0.00164	0.00365	0.67896
2014	102.63±0.68	113.89±0.73	0.00189	0.00461	0.72302
2012	104.20±0.59	119.43±0.76	0.00173	0.00431	0.85426
2011	105.47±0.60	115.91±0.74	0.00135	0.00306	0.75849
2009	103.99±0.56	120.02±0.77	0.00175	0.00408	0.82449
2008	104.69±0.32	119.99±0.36	0.00168	0.00347	0.7615
2006	104.28±0.61	143.85±0.92	0.00174	0.00361	1.25254
2004	105.88±0.60	132.39±0.84	0.0013	0.00368	1.03425
2002	106.72±0.62	158.20±1.01	0.0017	0.00387	1.56954
2001	112.01±0.34	154.38±0.47	0.00116	0.00339	1.45091
2000	108.29±0.62	122.16±0.78	0.00157	0.00324	0.75392
1998	106.83±0.61	136.94±0.87	0.00122	0.00299	1.04021
1997	113.76±0.34	145.44±0.44	0.00113	0.0034	1.30119
1996	105.58±0.60	154.95±0.99	0.00158	0.00365	1.39607
1994	105.54±0.32	117.37±0.49	0.00112	0.00367	0.58551
1993	104.59±0.59	118.75±0.76	0.00126	0.00301	0.5679
1990	104.71±0.32	119.97±0.36	0.00104	0.00335	0.53959
1983	97.31±0.56	108.07±0.69	8.10707E-4	0.00244	
1979	103.42±0.31	112.85±0.34	8.63511E-4	0.00331	
1977	99.96±0.57	107.51±0.69	6.57201E-4	0.0021	
**2019 No3**					
2018	102.45±0.55	117.32±0.13	0.00157	0.00353	0.8
2016	103.97±0.56	109.62±0.12	0.00227	0.0041	0.7

2015	103.91±0.56	115.39±0.13	0.0023	0.00389	0.7
2013	103.23±0.56	116.62±0.32	0.00194	0.00359	0.8
2012	104.85±0.56	117.45±0.13	0.00323	0.00377	0.8
2010	105.45±0.57	116.23±0.13	0.00187	0.00334	0.7
2008	106.75±0.57	120.69±0.13	0.00234	0.00321	0.8
2005	105.87±0.57	127.82±0.14	0.00189	0.00299	0.9
2003	109.22±0.59	125.24±0.14	0.00224	0.00286	0.9
2001	109.35±0.59	131.88±0.15	0.0014	0.00279	1.0
1999	108.07±0.58	136.97±0.15	0.00166	0.00283	1.0
1997	110.82±0.60	146.90±0.16	0.00201	0.00278	1.2
1994	104.67±0.56	125.38±0.14	0.00144	0.00268	0.7
1991	104.10±0.56	111.50±0.12	0.00193	0.00259	0.4
1988	104.55±0.56	111.50±0.12	0.00143	0.00207	0.3
1984	100.93±0.54	110.74±0.12	0.00153	0.00238	
1981	98.19±0.53	107.62±0.12	0.00104	0.00193	
1978	99.72±0.54	109.10±0.12	0.00152	0.00214	
1975	101.74±0.55	111.18±0.12	0.00112	0.00161	
1971	89.54±0.48	100.28±0.11	0.00102	0.00177	

This is S3 Table legend.
