# Supplementary figures and images for: What cooling pond sediments can reveal about 14C in nuclear power plant liquid effluents: Case study Lake Drūkšiai, Ignalina nuclear power plant cooling pond
Source: PLoS One. 2023 Oct 20;18(10):e0285531. doi: 10.1371/journal.pone.0285531 (PMC10588893; doi:10.1371/journal.pone.0285531)

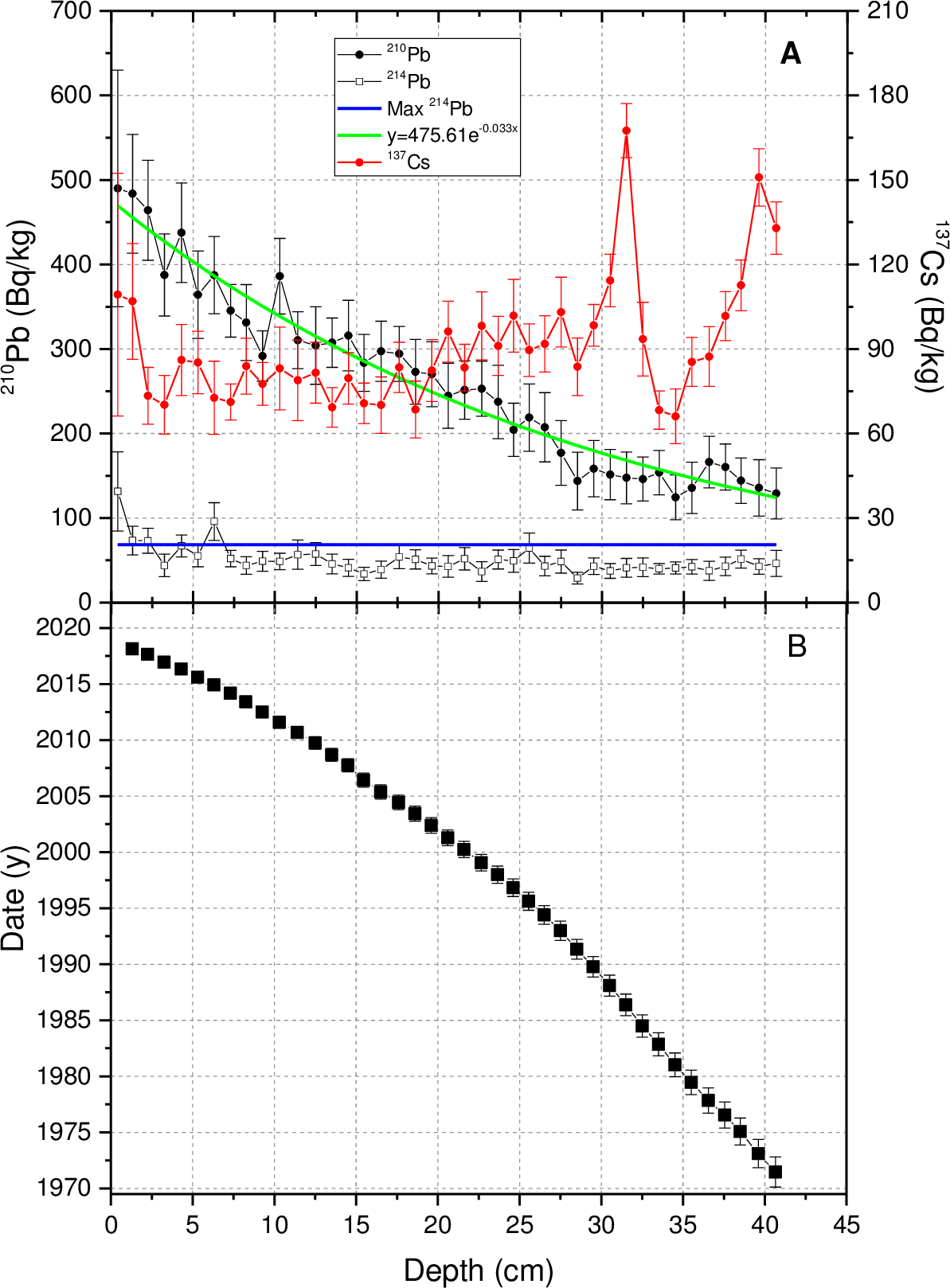

Supplement: S1 Fig — (TIF) [file pone.0285531.s005.tif]

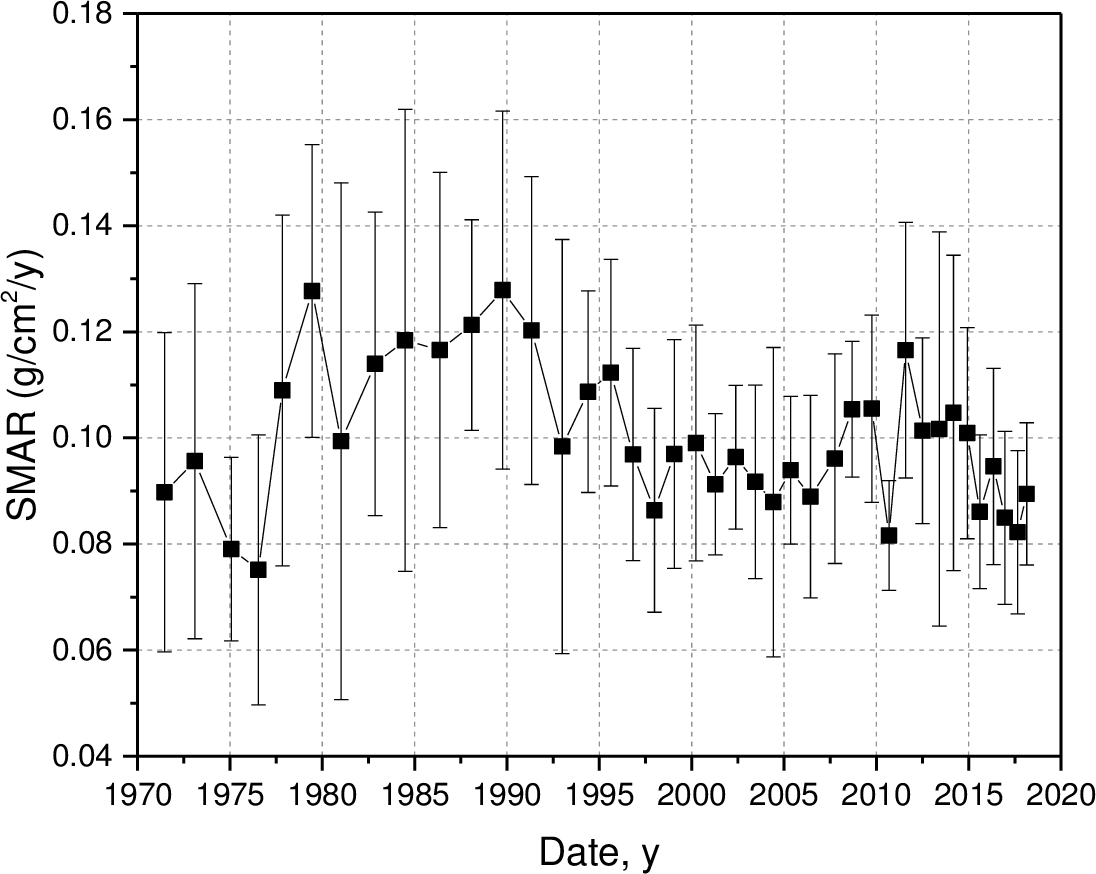

Supplement: S2 Fig — (TIF) [file pone.0285531.s006.tif]

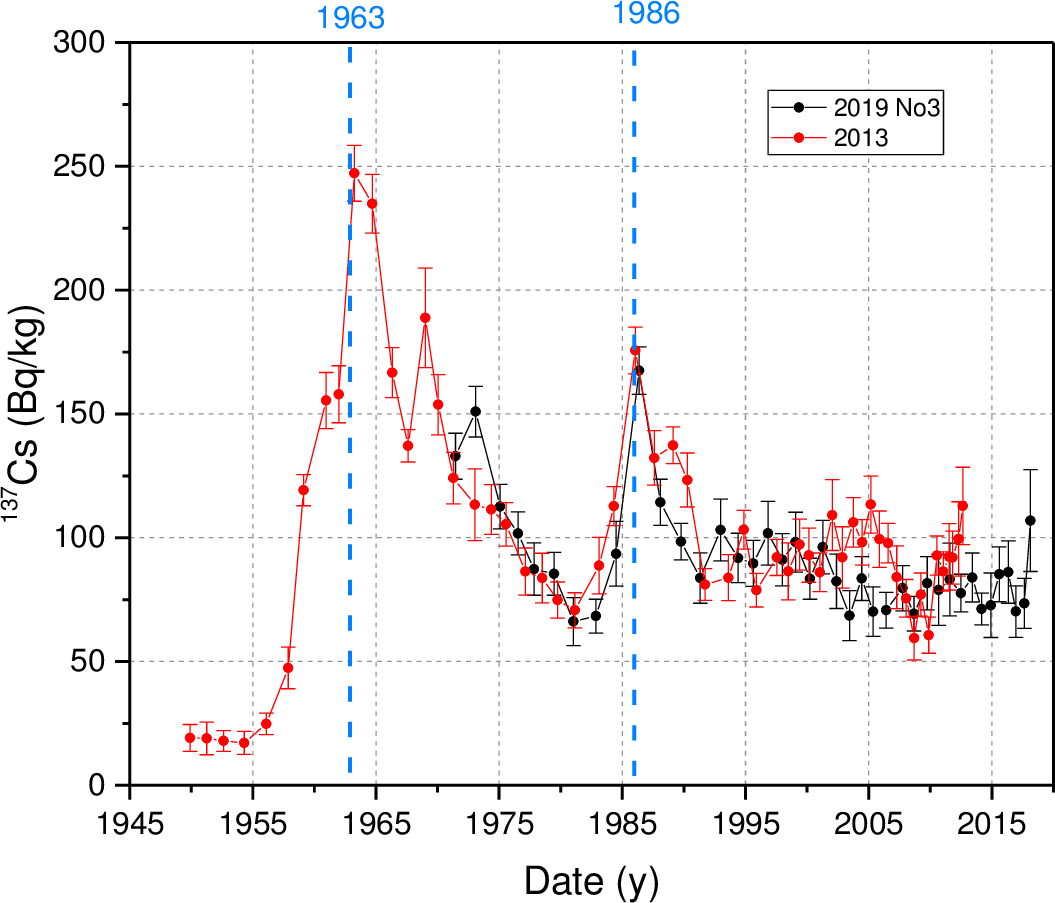

Supplement: S3 Fig — (TIF) [file pone.0285531.s007.tif]

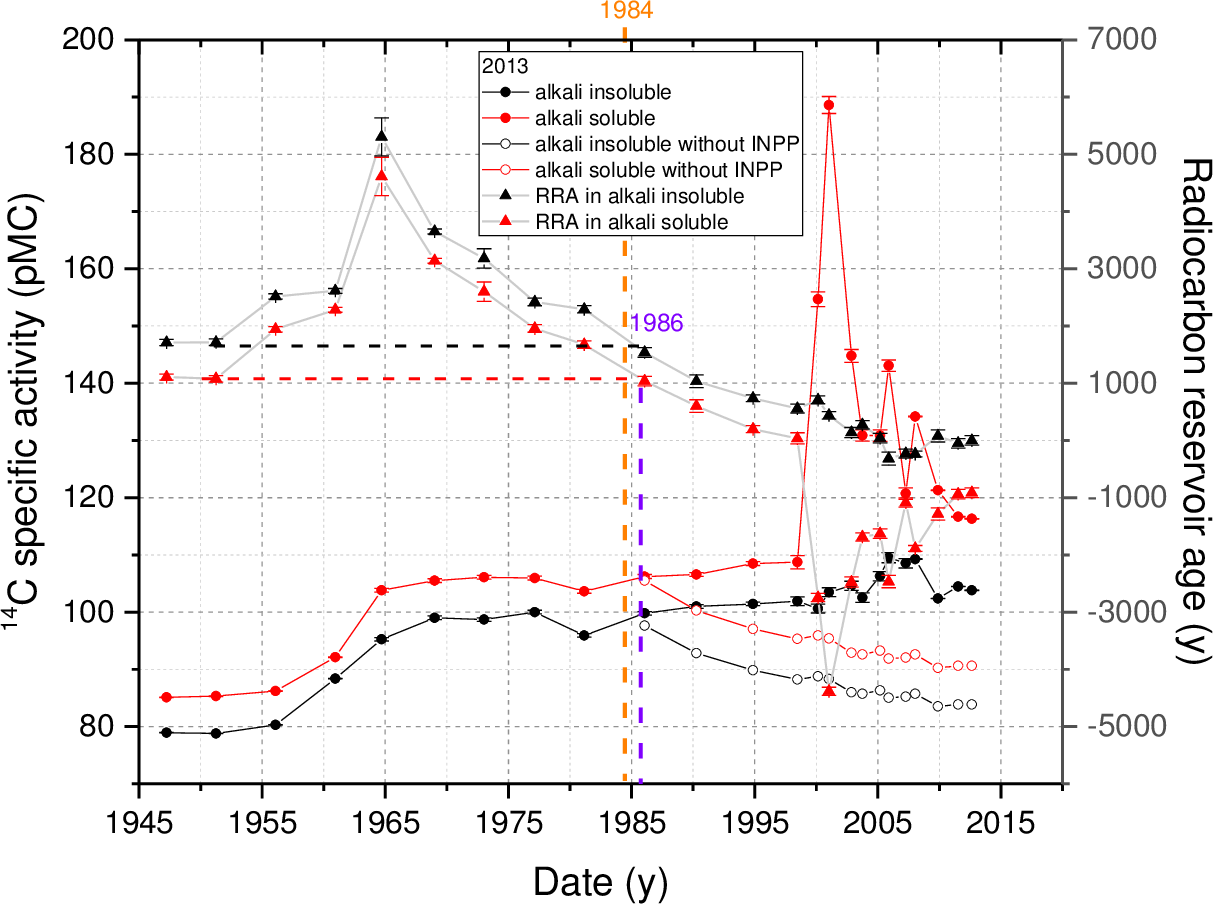

Supplement: S4 Fig — (TIF) [file pone.0285531.s008.tif]

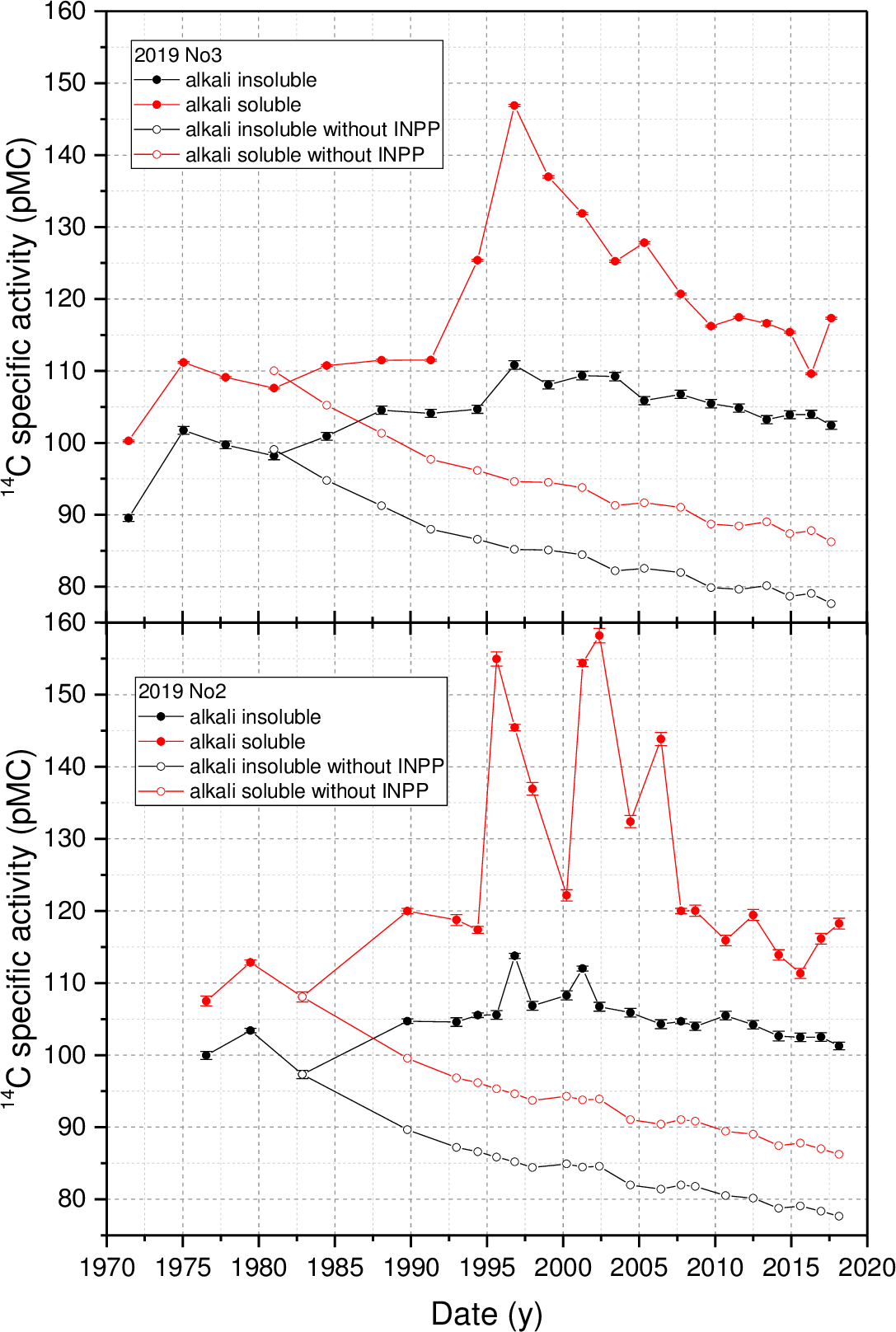

Supplement: S5 Fig — (TIF) [file pone.0285531.s009.tif]

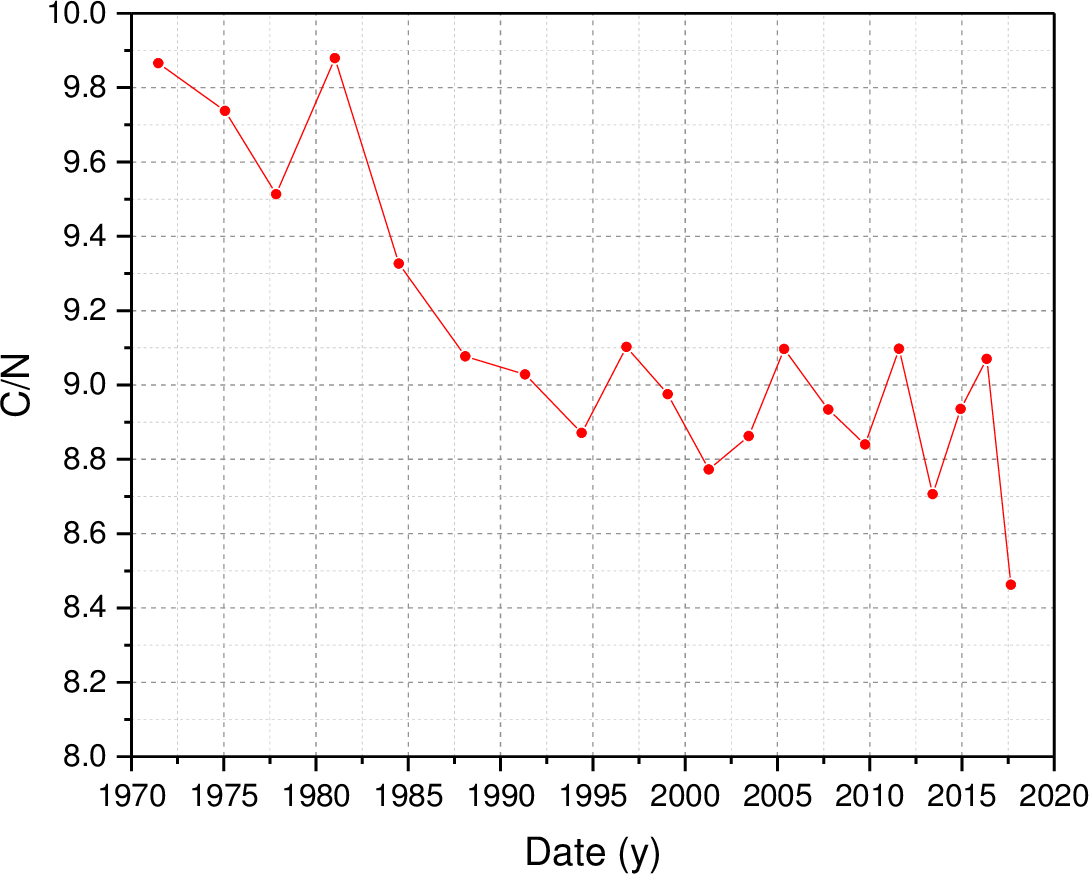

Supplement: S6 Fig — (TIF) [file pone.0285531.s010.tif]
